# Pervasive alterations of intra-axonal volume and network organization in young children with a 16p11.2 deletion

**DOI:** 10.1038/s41398-024-02810-5

**Published:** 2024-02-14

**Authors:** Anne M. Maillard, David Romascano, Julio E. Villalón-Reina, Clara A. Moreau, Joana M. Almeida Osório, Sonia Richetin, Vincent Junod, Paola Yu, Bratislav Misic, Paul M. Thompson, Eleonora Fornari, Marine Jequier Gygax, Sébastien Jacquemont, Nadia Chabane, Borja Rodríguez-Herreros

**Affiliations:** 1 Service des Troubles du Spectre de l’Autisme et apparentés, Département de psychiatrie, Lausanne University Hospital (CHUV), Lausanne, Switzerland; 2 Imaging Genetics Center, Mark and Mary Stevens Neuroimaging and Informatics Institute, Keck School of Medicine, University of Southern California (USC), Marina del Rey, CA USA; 3 Unité de Neurologie et neuroréhabilitation pédiatrique, Département femme-mère-enfant, Lausanne University Hospital (CHUV), Lausanne, Switzerland; 4 Department of Neurology and Neurosurgery, Montréal Neurological Institute, Montréal, QC H3A 2B4 Canada; 5 McConnell Brain Imaging Center, McGill University, Montréal, QC H3A 2B4 Canada; 6 Biomedical Imaging Center (CIBM), Department of Radiology, Lausanne University Hospital (CHUV), Lausanne, Switzerland; 7 Sainte Justine Hospital Research Center, Montréal, QC, Canada; 8 Department of Pediatrics, University of Montréal, Montreal, QC, Canada

**Keywords:** Autism spectrum disorders, Clinical genetics, Neuroscience

## Abstract

Reciprocal Copy Number Variants (CNVs) at the 16p11.2 locus confer high risk for autism spectrum disorder (ASD) and other neurodevelopmental disorders (NDDs). Morphometric MRI studies have revealed large and pervasive volumetric alterations in carriers of a 16p11.2 deletion. However, the specific neuroanatomical mechanisms underlying such alterations, as well as their developmental trajectory, are still poorly understood. Here we explored differences in microstructural brain connectivity between 24 children carrying a 16p11.2 deletion and 66 typically developing (TD) children between 2 and 8 years of age. We found a large pervasive increase of intra-axonal volume widespread over a high number of white matter tracts. Such microstructural alterations in 16p11.2 deletion children were already present at an early age, and led to significant changes in the global efficiency and integration of brain networks mainly associated to language, motricity and socio-emotional behavior, although the widespread pattern made it unlikely to represent direct functional correlates. Our results shed light on the neuroanatomical basis of the previously reported increase of white matter volume, and align well with analogous evidence of altered axonal diameter and synaptic function in 16p11.2 mice models. We provide evidence of a prevalent mechanistic deviation from typical maturation of brain structural connectivity associated with a specific biological risk to develop ASD. Future work is warranted to determine how this deviation contributes to the emergence of symptoms observed in young children diagnosed with ASD and other NDDs.

## Introduction

Rare pathogenic copy number variants (CNVs) confer high risk for neurodevelopmental disorders (NDDs) such as autism spectrum disorder (ASD) and schizophrenia. Recurrent hemizygosity (henceforth deletion) of a ∼600-kilobase (kb) region on the short arm of chromosome 16 (16p11.2 BP4-BP5) occurs at a frequency of ~3 in 10,000 and has attracted considerable attention as one of the most frequent genetic high-risk factors associated with ASD –identified in 1% of cases– and other NDDs [1–3]. The affected locus encompasses 29 protein-coding genes, several of them expressed in the brain [4]. Previous studies have associated the presence of a 16p11.2 deletion with attention deficit and hyperactivity disorder (ADHD), intellectual disability, language disorder and obesity [5–7], as well as an increased incidence of developmental coordination disorders and phonological processing [8, 9]. Intellectual quotient (IQ) is reduced in 16p11.2 deletion carriers by about 1.5 SD [10], and ~18% of them are diagnosed with ASD [6].

The study of brain structure in individuals sharing the same genetic factor –defined as a ‘genetic-first’ approach– allows the investigation of a specific neurobiological mechanism underlying NDDs, regardless of psychiatric diagnosis [11, 12]. A steadily increasing number of genetic-first neuroimaging studies of 16p11.2 deletion carriers have revealed neuroanatomical abnormalities affecting white matter (WM) tracts [13–18]. Two studies investigated WM microstructural integrity using diffusion tensor imaging (DTI) and tract-based spatial statistics (TBSS), demonstrating that 16p11.2 deletion children exhibit widespread increase of fractional anisotropy (FA), axial (AD) and mean diffusivity (MD), but no change in intra-axonal volume fraction (IAVF) [13, 16]. Nevertheless, the inherent limitations of TBSS restrict the inference of underlying functional and neurobiological implications [19], since voxel-based methods ignore the brain regions connected by a given WM tract, and large differences over a fascicle might be overlooked due to crossings with other unaffected tracts or tracts with opposite differences.

We aimed to overcome these limitations and characterize for the first time the structural connectome of children sharing a 16p11.2 deletion. We derived neuroanatomical signatures of brain microstructure and network organization in young children sharing the same biological mechanism predisposing for ASD and other NDDs. To that end, we used structural connectivity (SC) methods that evaluate microstructural integrity based specifically on the delineation of anatomical connections (i.e., streamlines or axonal trajectories reconstructed from diffusion MRI) [20]. Conventional SC analysis involves the reconstruction of anatomical pathways connecting gray matter areas, and the computation of adjacency matrices of various network metrics [21]. Several techniques have been proposed in recent years to estimate intrinsic microstructural features of the tissue, such as axonal density and diameter, by using multicompartment models. Here, we propose the combined use of the Convex Optimization for Microstructure Informed Tractography (COMMIT) [22], a novel framework to reestablish the link between tractography and tissue microstructure, together with the Stick-Zeppelin-Ball model [23]. SC data obtained using this combined methodology will provide streamline-specific quantitative microstructural metrics related to the fraction of restricted diffusion, conventionally linked to the IAVF [24]. Weighted network properties can then be acquired [25], and subsequently used to highlight differences in microstructural network topology [26].

Prior SC studies in ASD children have reported structural over-connectivity through an early and accelerated abnormal maturation of WM tracts [27, 28], which later decelerates and progresses into under-connectivity patterns in adolescents and adults [29]. Network inefficiencies in high-risk infants later classified as ASD have also been detected from 6 months onwards in regions involved in low-level sensory processing, propagating to higher-level cognitive regions through neurodevelopmental cascades at 12 and 24 months of age [30]. Heritable SC patterns have also been observed, as ASD boys have been found to share SC patterns with their fathers [31]. Despite these considerable advances in the neurodevelopmental trajectories of microstructural brain architecture in ASD children, their marked clinical heterogeneity limits their effect size and likely leads to poor replicability. By combining microstructural connectivity with a genetic-first approach, the present study overcomes heterogeneity of groups exclusively defined by behavioral criteria to dissect alterations of tissue microstructure and network organization in children sharing the same biological risk to develop ASD and other NDDs.

## Subjects and methods

### Participants

MRI data analyzed in this study was acquired from two different cohorts in Europe and North-America (Table 1): the Service des Troubles du Spectre de l’Autisme (STSA) at Lausanne University Hospital (CHUV), and the Searchlight cohort from the Simons Variation in Individuals Project (SVIP) [32]. Enrollment in the SVIP included referral by clinical genetic centers or active online registration. Families from the STSA cohort were directly referred to by their clinical geneticist who had initially established the presence of a proximal recurrent 600-kb (BP4-BP5; 29.6–30.2 Mb–Hg19) deletion at the 16p11.2 locus. TD children between 2 and 8 years of age were recruited from the general population through contact with local pediatricians and schools, between 2018 and 2021. Exclusion criteria included prematurity (<36 weeks of gestation), a known neurological condition or NDD, or a first-degree relative diagnosed with ASD. Each cohort was approved by its respective ethics review board, and written informed consent was obtained from STSA and SVIP participants’ legal representatives before their participation. This study was approved by the Commission Cantonale d’Ethique de la Recherche sur l’être humain (CER-VD, Switzerland), with the Project-ID 2018-00599.

**Table 1:** Demographics and main characteristics of the Simons Searchlight and STSA datasets used in our study.

	Simons Searchlight		STSA		Combined dataset		
	*16p11.2*	*TDs*	*16p11.2*	*TDs*	*16p11.2*	*TDs*	*p-val*
N	12	6	12	60	24	66	-
Age (SD) [range]	7.74 (0.8) [6.08-8.83]	7.37 (0.6) [6.50-8.08]	6.18 (1.7) [2.84-8.24]	5.62 (2.0) [2.30-8.98]	6.96 (1.5) [2.84-8.83]	5.78 (1.9) [2.30-8.98]	0.004
Sex (F/M)	3/9	3/3	3/9	31/29	6/18	34/32	**0.045**
NVIQ (SD)	92.3 (12.3)	102.5 (14.1)	93.2 (13.1)	111.3 (11.7)	92.7 (12.4)	110.5 (12.1)	**4e-7**
Motion mm (sd)	7.4 (5.1)	7.8 (6.0)	11.8 (8.2)	9.0 (4.8)	9.6 (7.1)	8.9 (4.9)	0.64

The final dataset included 24 children carrying a 16p11.2 CNV deletion and 66 TD children. 16p11.2 deletion carriers were older than TD children (6.96 vs. 5.78 years of age, t_89_ = 2.99, *p* = 0.004), and the proportion of males was higher in the genetic group (*X*^2^_(1,90)_ = 4.0, *p* = 0.045). There were no group differences regarding the amount of motion during the diffusion scan (t_89_ = 0.47, *p* = 0.64). We used Nonverbal IQ (NVIQ) as the outcome measure of cognitive level. We pooled NVIQ assessments using the Wechsler Preschool and Primary Scale of Intelligence (WPPSI-IV) [33], the 5th edition of the Wechsler Intelligence Scale for Children (WISC-V) [34], the Differential Ability Scales, Second Edition [35] and the Mullen Scales of Early Learning (MSEL) [36], according to child’s age and ability to comply with the tests. Children carrying a 16p11.2 deletion showed a 17-point decrease in NVIQ compared to TD children (t_89_ = −6.04, *p* = 4e-7).

### Data acquisition

The protocol used in the CHUV dataset is detailed here: (https://mcin.ca/research/neuroimaging-methods/acquisition-protocol). It includes two T1-weighted 3D brain volumes (0.9mm^3^ isotropic resolution, 256 × 256 × 192 matrix, GRAPPA acceleration factor of 4, TR/TE = 2300/2.32 ms, flip angle 8°), a T2-weighted volume (0.9mm^3^ isotropic resolution, 256 × 256 × 192 matrix, GRAPPA acceleration factor of 2, TR/TE 3200/408 ms), and a multi-shell diffusion-weighted imaging (DWI) sequence (1.7mm^3^ isotropic resolution, 150 × 154 × 87 matrix, slice acceleration factor of 6, TR/TE = 4100/71 ms, 7 directions with *b* = 300 s/mm^2^, 15 directions with *b* = 650 s/mm^2^, 30 directions with *b* = 1000 s/mm^2^, 60 directions with *b* = 2000 s/mm^2^, and 19 *b*0 volumes without diffusion weighting). A set of six *b*0 volumes was acquired with antero-posterior phase encoding (PE) while all other volumes were acquired with postero-anterior PE. Children were scanned without sedation in a Siemens 3 T Prisma scanner (Siemens, Erlangen, Germany) with a 64-channel coil. Scans were acquired in the early afternoon to favor naps in younger children. Parents could choose to administer melatonin to their child 30 minutes before the scan to help induce sleep/relaxation (6 or 10 mg for children below or above 6 years, respectively). Children were trained to remain still during a 15-min mock scan session prior to the real scan. In the real scan, children watched a movie of their choice through a mirror placed on the coil helmet.

The protocol of the SVIP dataset consisted of a T1-weighted volume (1mm^3^ isotropic resolution, 256 × 256 × 160 matrix, TR/TE = 2,530/1.64 ms, flip angle 7°) and a multi-shell DWI sequence (2mm^3^ isotropic resolution, slice dimension of 128×128 voxels, acceleration factor of 2, TE/TR = 80/10,000 ms for 30 directions with *b* = 1,000 s/mm^2^ and TE/TR = 119/13,900 ms for 64 directions with *b* = 3’000 s/mm^2^). Additional *b*0 volumes with no diffusion weighting were acquired at both TE/TR. Children were scanned at two different sites (University of California Berkeley and Children’s Hospital of Philadelphia), in 3 T Tim Trio Siemens MRI scanners (Siemens, Erlangen, Germany), using 32-channel head coils.

### Data preprocessing

**Fig. 1:**
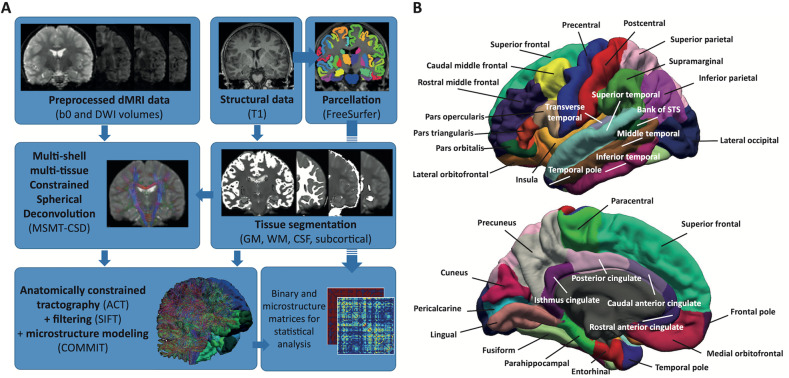
Pre-processing pipeline and cortex parcellation to generate SC matrices for each subject. **A** The pipeline includes segmentation and parcellation of the structural scan (T1), before registration to dMRI space. dMRI data is combined with tissue probability maps to compute MSMT Constrained Spherical Deconvolution. Tractography generates anatomically-valid streamlines that are then filtered using SIFT and used as input for the Convex Optimization for Microstructure Informed Tractography (COMMIT). **B** Cortical representation of the Desikan Atlas used in this study.

Figure 1A displays MRI preprocessing pipeline. Diffusion MRI data preprocessing involved denoising using Local Principal Component Analysis [37]; Gibbs ringing removal [38]; eddy current, susceptibility distortions (for subjects with opposite PE scans), subject movement correction with FSL’s v6.0.1 *Eddy* tool [39]; and outlier replacement and slice-to-volume correction [40]. Overall motion was computed as the norm of Eddy’s root mean square (RMS) output, which is the RMS of the distance between voxels in each DWI volume and the reference image. The preprocessed dMRI data was upsampled to 1mm^3^ isotropic resolution to improve tractography accuracy [41]. Field inhomogeneity correction was applied using the N4 algorithm as provided in ANTs [42]. Volumes in the SVIP dataset that were acquired at longer TE/TR were normalized to match the shorter TE/TR volumes to compensate for time differences between volumes (division by the averaged b0 volumes at longer TE/TR, and multiplication by the b0 at shorter TE/TR to keep anatomical contrast in b0 volumes). Cortical parcellation was performed using FreeSurfer (http://surfer.nmr.mgh.harvard.edu) in the T1-weighted volume. For subjects with opposite PE scans (i.e., corrected for susceptibility distortions), the average b0 volume was linearly registered from DWI to T1 space using a boundary-based cost function [43]. For subjects without opposite PE scans, the T1 was non-linearly registered to the average b0 volume using ANTs symmetric diffeomorphic image registration algorithm [44].

### Tractography

Tractography was performed using Anatomically Constrained Tractography (ACT) [45]. Five million streamlines per subject were generated using probabilistic tracking with second-order integration over FODs [46] combined with dynamic seeding from the WM FOD image [47], backtracking and ACT. ACT uses the tissue probability maps to ensure that reconstructed streamlines end in GM voxels. Dynamic seeding generated a set of streamlines (i.e., tractogram) that matched the CSD image, and backtracking re-tracks streamlines with poor anatomical termination. Tracked streamlines were further discarded if their length was longer than 250 mm. Quality control was assessed to detect registration issues, poor segmentation or parcellation by visually checking the tract density output from MRtrix.

### Computation of Intra-axonal volume and structural connectome

FreeSurfer Desikan-Killiany cortical [48] and Fischl’s subcortical [49] parcellations were converted to 85 ROIs using MRtrix’s label convert (Fig. 1B). The brain stem was added to the default 84 ROI conversion file to avoid discarding streamlines projecting to the spinal cord. The initial tractogram with 5 million streamlines was filtered down to 2.5 million streamlines using SIFT [50]. The contribution of each streamline to the dMRI signal was computed using COMMIT [22] and the three-compartment “Stick-Zeppelin-Ball” model [23]. This microstructure model accounts for restricted (i.e., water within axons, modeled as “sticks”), hindered (i.e., water between axons, modeled as “zeppelins”) and free water (i.e., partial volume with CSF, modeled as “balls”) compartments to assign weights that best fit the measured DWI signal. A 85×85 density weighted connectome was computed by summing the weights of the streamlines connecting pairs of ROIs, multiplied by the respective streamline lengths [26]. Streamlines were assigned to ROIs by radial search of up to 4 mm. No threshold was applied to remove weak connections, as recently recommended for weighted connectivity matrices [51]. Each subject’s total IAV was computed as the sum of the upper triangular connectivity matrix. Values in the connectivity matrix were divided by the total sum of weights, yielding the fraction of restricted signal allocated to connect each pair of ROIs, with respect to each subject’s brain.

### Statistical analysis

Shapiro-Wilk normality tests were conducted to confirm the normality distributions of the IAV and network metrics (*p* > 0.21). Group differences were assessed with an Analysis of CoVariance (ANCOVA) model in R (v4.2.1) [52]. We used the group factor (16p11.2 or TD) as the main variable, and age, sex, scanning site, and overall motion during the scan as covariates. Levene test was used to assess equality of variance. A Generalized Additive Models for Location, Scale and Shape (GAMLSS) [53, 54] was also fitted using the Python PCNtoolkit [55], to estimate the typical developmental growth of total IAV as a function of age with sex, motion and site as covariates. The effect of age was modeled using 2nd order B-splines with 3 knots, and parameters were fitted using bayesian linear regression. This normative model was computed using TD data only. We tested for a significant group effect as well as for a significant group*age interaction in total IAV. The same procedure was used to study developmental trajectories of global network metrics (see below).

To further explore how IAV differences might manifest in pairwise –ROI to ROI– connectivity strengths, we tested for SC differences in each of the 3’570 possible connections. Raw IAV values were compared, as well as IAVF after normalizing for total IAV. P-values were corrected using False Discovery Rate (FDR). The Pearson correlation between fascicle length and effect size was computed to assess whether group differences were associated with short-range or long-range connections. We tried to determine which functions were affected by labeling nodes according to the 7 functional labels of Yeo atlas (visual, somatomotor, dorsal attention, ventral attention, limbic, frontoparietal, and default mode network (DMN)) [56], combined with additional labels for cerebellum, basal ganglia and brainstem. Intra-functional differences were estimated by fitting an ANCOVA model to the sum of weights of connections linking ROIs belonging to the same network. Between-network differences were obtained by summing the weights of connections linking ROIs belonging to pairs of different networks.

We also tested for differences in the following network node metrics: node strength, node closeness, node betweenness, node efficiency, node clustering coefficient, and node participation coefficient. We used weighted formulations available in the iGraph library [57] (see Supplementary Methods). Node participation coefficients were computed using Yeo’s functional parcellation of the 85 ROIs, plus cerebellum, basal ganglia and brainstem as additional labels. P-values for node features were corrected for multiple comparisons using FDR across the 85 nodes for each metric. To summarize nodal differences, we performed a probabilistic PCA on the Cohen d values using *pcaMethods* R library. We used probabilistic PCA since the effect-size for betweenness could not be computed for 6 nodes (1.2% of the data). Betweenness could not be computed for these 6 nodes because they did not belong to any shortest path, resulting in the comparison of two null vectors. Two principal components (PCs) were generated and their values projected on the pial surface for visual representation.

## Results

### Group differences and normative modeling of total IAV

**Fig. 2:**
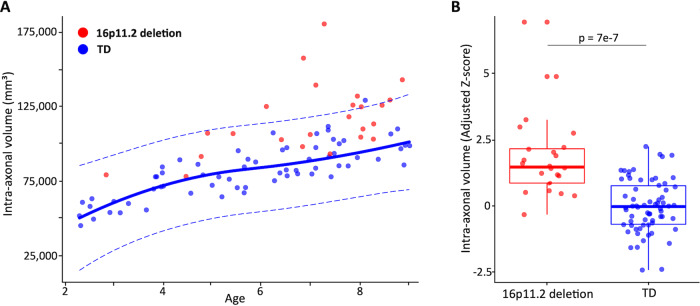
Normative model and group differences for total IAV. **A** The main curve represents the mean intra-axonal volume as a function of age, and dotted lines the 2.5 and 97.5 percentiles respectively. The area between the two dotted lines represents values expected to cover 95% of the typically developing population. TD values are reported in blue and 16p11.2 deletion carriers in red. Values were adjusted for the effect of sex, motion and site as estimated by the GAMLSS model. **B** Histogram of IAVF values for the two groups, z-scored with respect to the mean and variance of the TD group after adjusting for age, sex, motion and site. The ANCOVA showed that 16p11.2 carriers had a significant increase in IAVF with respect to TDs (F-statistic = 21.18 on 6 and 83 degrees of freedom). Cohen’s d on the intra-axonal volume adjusted for age, sex, motion and site was 1.55.

Figure 2A shows the developmental trajectory for global IAV in TD children as estimated from the GAMLSS fit. All children carrying a 16p11.2 deletion exhibited global IAV values above the median TD curve. As a result, 16p11.2 deletion carriers present a significant increase in total IAV (127’458 mm^3^ vs. 107'034 mm^3^, F_6,83_ = 21.18, t_(83)_ = 5.36, *p* = 7e-7, *d* = 1.55, Fig. 2B). We did not find a significant interaction with age (F_7,82_ = 18.3, t_(82)_ = −1.01, *p* = 0.32). The variance of IAV was significantly higher in 16p11.2 deletion children (Levene test, *p* < 0.05).

### Mapping of IAV increase in 16p11.2 deletion across structural and functional networks

**Fig. 3:**
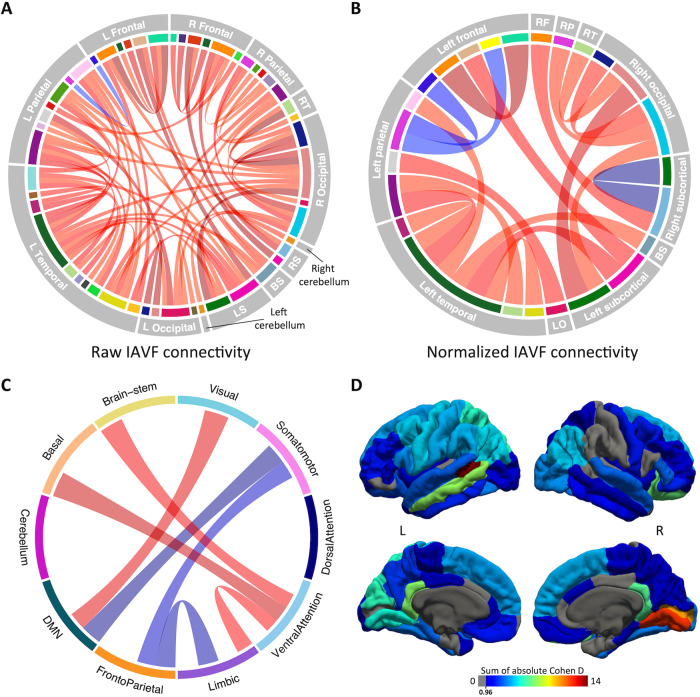
Distribution of IAV differences across brain connections. Chord diagrams show regional raw (**A**) and normalized (**B**) differences in IAV. Chord color corresponds to the Cohen’s d value obtained from the raw IAV, adjusted for age, sex, motion and site. Brain regions are ordered by hemisphere and lobe. R right, L left, RS right subcortical, BS brain stem, LS left subcortical, RF right frontal, RP right parietal, RT right temporal, BS brain stem, LO left occipital. **C** Chord diagram illustrating significant group differences in IAVF between functional networks. Network labels were taken from Yeo atlas (visual, somatomotor, dorsal attention, ventral attention, limbic, frontoparietal, and default mode networks; Yeo et al. 2011), combined with additional labels for cerebellum, basal ganglia and brainstem. DMN Default Mode Network. **D** Cortical regions labeled according to the sum of Cohen’s d computed from the normalized connectivity matrix. For readiness, only the connections with the 20% highest absolute effect sizes are shown. L left, R right.

Regionally, raw IAV differences in 317 out of 3’570 individual connections survived FDR correction (Fig. 3A). Specifically, 16p11.2 deletion children showed higher IAV in 293 out of these 317 statistically significant connections. Effect size was significantly correlated with fascicle length (t_(3558)_ = 2.62, *p* = 0.001), though with low correlation coefficient (rho = 0.04). When normalized by the total IAV, 97 out of 3’570 connections survived FDR correction, 65 of which were associated with increased IAVF in 16p11.2 carriers (Fig. 3B). Top 10 connections with largest effect size are reported in Table 2; most of them involved intra-hemispheric connections in the left hemisphere, and both cortico-cortical and subcortical-cortical connections. Group-wise mean IAVF values, Cohen’s d and p-values for all statistically significant connections are available in Supplementary Tables S1 (raw) and **S2** (normalized).

**Table 2:** Connection-wise normalized SC differences between 16p11.2 deletion and TD children.

Connection	Mean in 16p11.2	Mean in TDs	Cohen’s d	p-value
Right thalamus - Right caudate	5.04e-03	6.93e-03	−1.95	7e-06
Left lateral orbitofrontal - Left superior frontal	4.69e-04	2.29e-04	1.83	2e-05
Left thalamus - Right lingual	2.38e-04	8.86e-05	1.71	8e-05
Left banks STS - Left putamen	1.19e-04	4.75e-05	1.57	4e-04
Left banks STS - Left isthmus cingulate	1.69e-05	5.66e-06	1.56	4e-04
Left pars opercularis - Left putamen	1.59e-03	9.11e-04	1.47	1e-03
Left inferior parietal - Left precentral	3.94e-04	8.63e-04	−1.46	1e-03
Left cuneus - Left precuneus	1.14e-03	7.18e-04	1.44	1e-03
Right Caudate - Right lateral orbitofrontal	7.18e-04	3.06e-04	1.43	1e-03
Right lateral occipital - Right pericalcarine	1.78e-03	1.26e-03	1.42	2e-03

Mean SC values were computed from normalized values. Cohen’s d were computed after adjusting for the effect of age, sex, motion and sit.

Figure 3C depicts statistically significant differences in IAVF between several functional networks, according to labels of Yeo’s atlas. Inter-network IAVF in 16p11.2 deletion children was significantly increased between visual and DMN, as well as between ventral attention and brain stem, basal ganglia and limbic networks. On the other hand, IAVF was found to be reduced between frontoparietal and both limbic and somatomotor networks, as well as between somatomotor and DMN. Both intra and inter-functional differences in IAVF are summarized in Table 3. Finally, Fig. 3D shows the sum of absolute effect sizes computed from the normalized IAVF connectivity matrix. We observed the largest cumulated effect size in the left banks of the STS. The left middle temporal cortex, bilateral isthmus cingulate, right lateral orbitofrontal, pericalcarine and lingual cortices presented mild cumulated effect size.

**Table 3:** Connections within and between functional networks with significant IAVF differences between 16p11.2 deletion and TD children.

Functional connection	Mean in 16p11.2	Mean in TDs	Cohen’s d	p-value
Visual - Visual	8.33e-02	7.54e-02	1.41	3e-04
Basal - Basal	9.58e-02	1.08e-01	-1.14	5e-03
Somatomotor - DMN	4.77e-02	5.16e-02	-1.06	7.9e-03
Limbic - FrontoParietal	4.81e-03	6.59e-03	-0.99	0.01
VentralAttention - Basal ganglia	3.13e-02	2.87e-02	0.95	0.01
Somatomotor - FrontoParietal	6.62e-03	8.18e-03	-0.93	0.02
Visual - DMN	3.13e-02	2.95e-02	0.90	0.02
VentralAttention - Brain-stem	3.93e-03	3.34e-03	0.86	0.02
Cerebellum - Cerebellum	5.21e-02	6.2e-02	-0.81	0.04
VentralAttention - Limbic	9.91e-03	9.44e-03	0.81	0.04

Mean values were computed from the sum of IAVF belonging to the respective connections. Cohen’s d were computed after adjusting for the effect of age, sex, motion and site.

### Group differences and developmental trajectories of global structural network metrics

**Fig. 4:**
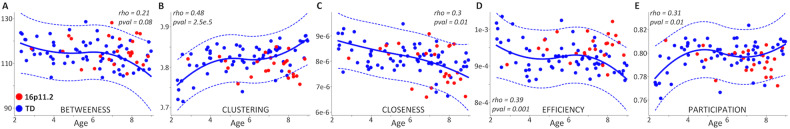
Age-related normative changes in global network metrics. The scatterplots show betweeness (**A**), clustering coefficient (**B**), closeness (**C**), efficiency (**D**) and participation coefficient (**E**), as estimated in TD children (blue) by GAMLSS univariate distributional regression modeling. The full line depicts the estimated median, whereas the dotted lines represent the 2.5 and 97.5 percentiles, respectively. Metric values for 16p11.2 deletion children were later added in red. Each metric is adjusted for sex, motion and site. All parameters were fitted using bayesian linear regression. Rho Pearson provides a measure of linear correlation between age and metric values. Global strength was not included as its normalization with respect to total IAV results in constant value for all subjects.

Global estimates of the developmental growth in TD children for the analyzed network metrics are presented in Fig. 4. Global efficiency presented an overall decrease over age with a plateau between 5 and 7 years (rho = 0.39, *p* = 0.001), whereas global closeness exhibited a steadily constant decrease through childhood lifespan (rho = 0.3, *p* = 0.01). Clustering (rho = 0.48, *p* = 2.5e-5) and participation (rho = 0.31, *p* = 0.01) coefficients showed an overall increase with again a short plateau between 5 and 7 years. The group comparison showed that children with a 16p11.2 deletion exhibited a significant increase in weighted global efficiency (9.5e-4 compared to 9.23e-4 for TDs, *d* = 0.80, t_(83)_ = 2.76, *p* = 0.007) and global betweenness (116 compared to 114 in TDs, *d* = 0.64, *t*_(83)_ = 2.21, *p* = 0.03).

### Mapping and latent dimensions of differences in structural network organization

**Fig. 5:**
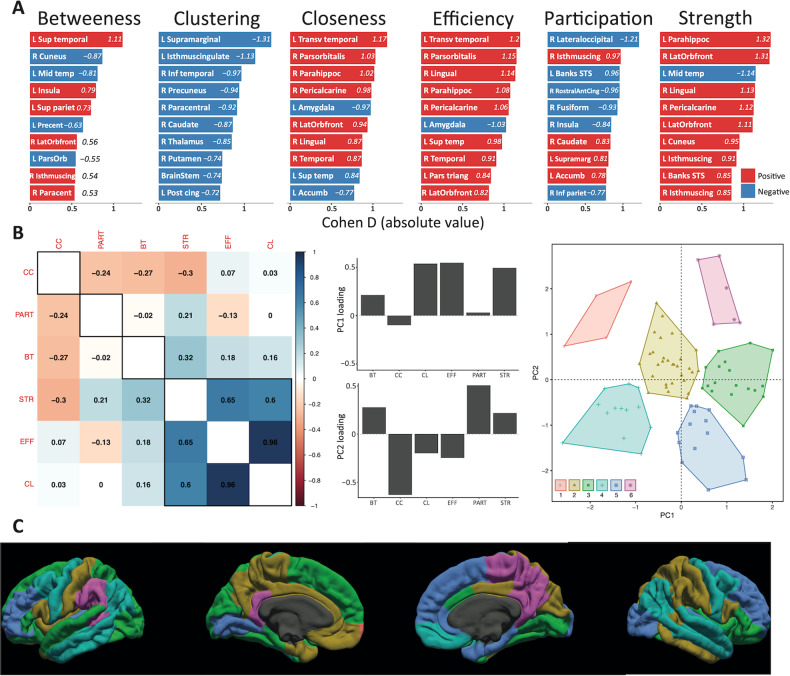
Distribution and latent dimensions of differences in structural network properties. **A** Node ranking according to absolute Cohen’s d, for each of the six network metrics used in the study. Red indicates positive effect sizes, that is, higher metric values in 16p11.2 children than TD, and vice versa for blue (negative) effect sizes. **B** Correlation matrix between the six network metrics used in the study, grouped by hierarchical clustering (left). Loadings of PC1 and PC2 for the six different metrics (middle). K-means clustering of the PC1 and PC2 scores for the 85 brain nodes used in our study (right). BT betweenness, CC clustering coefficient, CL closeness, EFF efficiency, PART participation coefficient, STR strength. **C** K-means clusters projected on the pial surface. Each cortical region is colored according to the clusters reported in (**B**).

Figure 5A shows the top 10 regions with largest effect size for each of the six network metrics. Out of 85 ROIs considered in this study, 28 had a significant group difference in at least one of the six nodal network metrics. Largest differences for each metric included higher nodal strength in the left parahippocampal cortex (*d* = 1.32, *t*_(83)_ = 4.57, *p* = 8.6e-4) and higher efficiency in the left transverse temporal cortex of 16p11.2 deletion children (*d* = 1.20, *t*_(83)_ = 4.13, *p* = 0.005), together with higher betweenness in the left superior temporal cortex (*d* = 1.11, *t*_(83)_ = 3.82, *p* = 0.02) and higher closeness in the left transverse temporal gyrus (*d* = 1.17, *t*_(83)_ = 4.04, *p* = 0.01). Conversely, TD children exhibited higher clustering coefficient in the left supramarginal cortex (*d* = −1.31, *t*_(83)_ = −4.54, *p* = 0.002) and higher participation coefficient in the right lateral occipital cortex (*d* = −1.21, *t*_(83)_ = −4.19, *p* = 0.006). The cortical Cohen D map for all significant topological differences in each of the six network metrics is illustrated in Supplementary Figure 1. Group mean, Cohen’s d and p-values in each network metric for all regions with significant group differences are summarized in Supplementary Table S3.

Finally, a PCA on Cohen’s d values derived from weighted network metrics delineated the latent dimensions explaining the abovementioned regional differences. The two PCs captured 43% and 24% of the variance, respectively. The first latent dimension (PC1) was highly loaded by closeness, efficiency and nodal strength, while PC2 was positively loaded by participation coefficient and negatively loaded by clustering coefficient (Fig. 5B). The ‘silhouette’ approach determined that 6 clusters best summarized the distribution of brain nodes in the PC1-PC2 plane. Figure 5C depicts the topological distribution of PC1-PC2 clusters. Supplementary Figure S2 shows PC1 and PC2 scores for each region, represented on the pial surface. Regions with highly positive PC1 scores –indicating large positive effect sizes (16p11.2 > TD) on closeness, efficiency and nodal strength– included the right lateral orbitofrontal, lingual, and pericalcarine gyrus. Conversely, most negative PC1 scores corresponded to the left middle temporal, amygdala, and accumbens area. With respect to PC2, the right temporal pole, parahippocampal, and lateral occipital gyrus presented highly positive scores, whereas most negative values included the left supramarginal, isthmus cingulate and right caudate.

## Discussion

In this study, we characterized brain tissue microstructure and patterns of network organization in young children sharing a deletion of the 16p11.2 locus, a genetic risk factor predisposing for ASD and other NDDs. Our results show striking differences compared to typical neurodevelopmental trajectories of WM maturation. Children with a 16p11.2 deletion exhibit a large pervasive increase of intra-axonal volume, widespread over a high number of WM connections irrespective of fascicle length. Such microstructural alterations also led to significant changes in brain network organization. Several functional networks, mainly associated with language, motricity and socio-emotional behavior, were affected. Global developmental trajectories of specific network properties deviated from typical brain development already in early childhood. Our findings add to recent direct neuroanatomical observations in 16p11.2 animal models, and pinpoint alterations in white matter maturation underlying a specific biological risk associated with ASD.

### Neurobiological interpretations of altered tissue microstructure

Just as the size and capacity of roadways can regulate the flow of traffic, microstructural properties of WM tracts determine the amount and quality of information transmitted between different brain regions [58]. Here we observed an overall increase of total IAV in children carrying a 16p11.2 deletion, a putative neuroanatomical underpinning of the widespread increase of WM volume reported in other volumetric studies [15, 17, 59]. Previous work also hinted at a unique signature of WM microstructural integrity in 16p11.2 deletion children, namely the joint increase of FA, AD and MD compared with TD children [13, 16]. This signature differs from the usual decrease in FA and increase in mean and radial diffusivity observed in ASD and other NDDs [60, 61]. An increase in IAV –as observed in our study– would be compatible with an increase in FA, MD and AD [62, 63], and could represent a neuroanatomical deviation from typical brain maturation specific to the pathophysiology of 16p11.2 deletion. Also, a higher IAV can be associated with a higher number of axons and/or a larger axonal diameter, both of which could explain increased FA, MD, and AD. Unfortunately, our diffusion MRI protocol does not allow to disentangle between these two neuroanatomical substrates; for that, preclinical MRI scanners with gradient strengths above 300 mT/m would be required [64, 65].

Despite the significant evolutionary distance between rodents and humans, the alignment between our extensive increase of IAV and the analogous widespread FA mapping in the 16p11.2 deletion mice serves as compelling evidence supporting the translational validity of the mouse model [66]. Bertero and colleagues suggest that altered feedback from the cortex leads to abnormal maturation of thalamo-cortical projections, a mechanism previously described by Thompson et al. (2016) [67]. Similarly, other studies demonstrated shortened cell cycles of neuronal progenitor cells in 16p11.2 mice, leading to increased number of cortico-thalamic neurons [68, 69]. All these neuroanatomical observations might be concomitant with the abnormalities in microstructural tissue integrity that we found in 16p11.2 deletion children. One possibility is that the imbalance between the number of cortico-thalamic and cortico-cortical neurons due to altered cell cycles could modulate synaptic function and affect neuronal pruning, possibly deviating axonal growth and projections to secondary regions. Nevertheless, the interpretation of the neurobiological alterations underlying changes in WM tissue microstructure require conclusive validation by histological or tracing methods.

Our study showed that IAV was largely increased in structural connections between visual and DMN, while decreased within basal ganglia and between somatomotor and DMN. These findings are consistent with previous evidence of reduced long-range functional coupling between temporal and parietal regions [66]. We found the largest differences on tissue microstructure in brain regions generally implicated in language and socio-emotional behavior, such as the superior and the transverse temporal gyri [70–72], as well as motor skills associated with altered microstructural architecture of the bilateral precentral gyri [73]. However, these are diverse functions that are complex and heterogeneous, and we do not imply that our findings are specific to the 16p11.2 locus. Indeed, similar major cortico-cortical connections exhibited large effects on WM microstructure in 22q11.2 deletion carriers [74]. These observations might indicate a convergence in altered tissue microstructure across distinct genetic risk factors [12], but whether these shared structural differences represent analogous underlying molecular mechanisms remains to be clarified. A recent study shows that the mapping of CNV-related structural alterations aligns well with the spatial distribution of gene expression in the adult brain [75]. Therefore, developmental trajectories of gene expression in the brain could provide valuable insights in understanding the altered structural connectome of 16p11.2 deletion carriers.

Likewise, the intersection between the regions with the largest increase in IAV and a meta-analysis encompassing all Axis I psychiatric diagnoses in DSM-IV-TR might offer clues about the pathophysiological patterns contributing to the risk of psychiatric diagnoses associated to the 16p11.2 deletion [76]. Furthermore, this partial overlap supports the additive model account, by which CNV-related structural alterations confer a risk, but may not be necessarily associated with a psychiatric diagnosis [77]. Based on this premise, the presence of a neuropsychiatric disorder may require supplementary brain alterations and/or other contributing factors [78]. The fact that these alterations in tissue microstructure are not strictly dependent on a specific clinical diagnosis suggests that a deletion of the 16p11.2 locus mediates risk by modulating the continuum of normative brain structure, as would be expected as well for intermediate phenotypes related to behavior and cognition [79]. As opposed to idiopathic conditions, the CNV effect size that we report on IAV is comparable to those of cognitive and behavioral phenotypes, suggesting that neuroimaging traits might serve as mediators for cognitive and behavioral features. Nonetheless, our study does not allow to establish causal links between the WM neuroanatomical abnormalities and corresponding functional correlates. Further work combining structural quantitative tractography, multimodal neuroimaging and task-based paradigms is warranted to reveal causal associations between altered WM microstructural integrity and behavioral correlates.

### Implications of altered development of network topology in 16p11.2 children

Beyond individual WM tracts, there is also a growing interest in characterizing structural networks during early brain development and quantifying different information-transfer properties [80]. Among these properties, indices of local and global efficiency characterize the ease of information flow at the local neighborhood and global system levels, respectively [81]. These structural networks are altered in neuropsychiatric disorders, and changes in the number and/or the diameter of axons are expected to influence the conduction velocity of action potentials [82], therefore disrupting timely orchestrated communication delays and synchronicity between brain regions [83]. The reconstructed structural networks of young children carrying a 16p11.2 deletion exhibited both global and local estimates of altered structural network topology. We observed a significant increase in weighted global efficiency and betweenness, potentially reminiscent of the developmental disconnection theory, already suggested for ASD and proposing a decreased long-range integration accompanied by local overconnectivity and decreased functional specialization in the brain [84].

Network maturation in the first 2 years after birth generally consists of increased efficiency and network integration, decreased network segregation and some changes in modularity, with most major hubs and modules in place by 2 years of age. Remarkably, our young TD children between 2 and 8 years of age exhibited a slight decrease of network integration over time –as illustrated by global efficiency–, while segregation increased –as illustrated by the global clustering coefficient [25]. Certain properties of centrality such as betweenness and closeness decreased between 2 and 8 years, while participation coefficient increased. In other words, the average brain region typically becomes less and less central between 2 and 8 years of age, and its average connectivity to regions outside its functional cluster increases. We barely identified significant interactions with age on structural network metrics, suggesting that most microstructural alterations in 16p11.2 deletion carriers are already present at an early age. Recently, altered efficiency has been observed in low-level sensory processing areas at already 6 months of age in babies at risk for ASD [30]. The same study also shows that deviation from typical development in toddlers later diagnosed with ASD leads to reduced efficiency in higher-level frontal cortices at 12 and 24 months. This postero-anterior cascade follows GM and WM maturation traits described in typical development, where primary sensory areas mature before association and higher-level regions [85–87]. A comparable process in 16p11.2 deletion carriers could explain the larger effect that we observed in visual networks, the basal ganglia and somatomotor areas. As a result, microstructural differences in higher-level associative regions should increase with age. A longitudinal study is warranted to confirm this hypothesis, already pinpointed in previous studies reporting the deviation of early developmental trajectories of GM maturation in 16p11.2 deletion and duplication carriers [59].

### Limitations

Diffusion tractography has recently been shown to contain high rates of false positive connections [88]. Our study is not safe from false-positive connections, even though ACT, SIFT, and COMMIT were used to reduce false-positives and increase the neuroanatomical accuracy of the reconstructed tractograms [22, 45, 50]. Part of the differences in weighted connectivity metrics could be due to methodological factors, such as T2-dependent properties which are not considered in the Stick Zeppelin Ball model. Nonetheless, the abovementioned restraints do not compromise the reliability of our findings, since the convex formulation of COMMIT framework effectively combines local tissue properties with the versatility of classical fiber-tracking algorithms, providing optimal tractograms that closely resemble known brain anatomy. T2-dependent effects on network metrics were minimized using minimum TE to maximize SNR and long enough TRs to ensure no gradient heating effects. For each diffusion-weighting combination we acquire additional *b* = 0 volumes with no diffusion weighting to correct for T1 and T2 dependence. Finally, the sample size of 16p11.2 deletion carriers is limited by the relatively rare frequency of CNVs in this locus (~1/2000). However, our results provide robust estimates for CNV effect sizes on brain microstructure. Our sample size was chosen based on previous neuroimaging studies on 16p11.2 deletion carriers, enough to detect similar effect sizes to those observed in these preceding investigations. Further growth of public datasets will surely provide more statistical power, but our sample is the largest available to assess microstructural connectivity and adequate to detect the large effects associated with 16p11.2 deletion, greatly reducing the probability of spurious findings.

## Conclusion

We characterized for the first time the structural connectome of 16p11.2 deletion carriers. We show that the brain of young children carrying a 16p11.2 deletion exhibits an altered network organization, as well as a widespread increase of IAV, suggesting a putatively altered microstructural architecture and synaptic function such as in 16p11.2 mice models. Our findings provide evidence of a prevalent mechanistic deviation of typical maturation of brain microstructure associated with a specific biological risk to develop ASD and other NDDs. Future work should assess how early individual neuroanatomical alterations relate to ASD core symptomatology. Tentatively, one possibility is that alterations in structural network organization were associated to early warning ASD-related phenotypic manifestations. Identifying neuroanatomical predictors of the emergence of atypical developmental paths, in precursors such as sensorimotor function, could lead to earlier signaling of at-risk children and improve recommendations for timely intervention and parental support.

### Supplementary information

Supplemental Material

Supplemental Table S1

Supplemental Table S2

Supplemental Table S3

## Data Availability

The STSA dataset is available upon reasonable request to borja.rodriguez-herreros@chuv.ch. Approved researchers can obtain the SVIP dataset by applying at https://base.sfari.org. The code used for the analysis is publicly available at https://github.com/stsa-research.
