# Supplementary material for: Pervasive alterations of intra-axonal volume and network organization in young children with a 16p11.2 deletion
Source: Transl Psychiatry. 2024 Feb 14;14:95. doi: 10.1038/s41398-024-02810-5 (PMC10866898; doi:10.1038/s41398-024-02810-5)
Supplement: Supplementary file 1 — Supplemental Material [file 41398_2024_2810_MOESM1_ESM.pdf]

Supplemental material

Pervasive alterations of intra-axonal volume and network organization in young children with a 16p11.2 deletion

Running title: *Altered structural connectome of 16p11.2 deletion*

Anne M. Maillard[1†]PhD, David Romascano[1†]PhD, Julio E. Villalón-Reina[2] PhD, Clara A. Moreau[2] PhD, Joana M. Almeida Osório[1] PhD, Sonia Richetin[1] MSc, Vincent Junod[3] MSc, Paola Yu[1] MSc, Bratislav Misic[4,5] PhD, Paul M. Thompson[2] PhD, Eleonora Fornari[6] PhD, Marine Jequier Gygax[1] MD, Sébastien Jacquemont[7,8§] MD, Nadia Chabane[1§]MD PhD and Borja Rodríguez-Herreros[1§] PhD

†These authors contributed equally to this work.

§These authors contributed equally to this work.

[1]Service des Troubles du Spectre de l'Autisme et apparentés, Département de psychiatrie, Lausanne University Hospital (CHUV), Lausanne, Switzerland

[2]Imaging Genetics Center, Mark and Mary Stevens Neuroimaging and Informatics Institute, Keck School of Medicine, University of Southern California (USC), Marina del Rey, CA, USA

[3]Unité de Neurologie et neuroréhabilitation pédiatrique, Département femme-mère-enfant, Lausanne University Hospital (CHUV), Lausanne, Switzerland

[4]Department of Neurology and Neurosurgery, Montréal Neurological Institute, Montréal, QC H3A 2B4, Canada

[5]McConnell Brain Imaging Center, McGill University, Montréal, QC H3A 2B4, Canada

[6]Biomedical Imaging Center (CIBM), Department of Radiology, Lausanne University Hospital (CHUV), Lausanne, Switzerland

[7]Sainte Justine Hospital Research Center, Montréal, Canada

[8]Department of Pediatrics, University of Montréal, Montreal, Canada

Correspondence to: **Borja Rodríguez-Herreros, PhD**
Service des Troubles du Spectre de l'Autisme & apparentés
Centre Cantonal de l'Autisme
Les Allières, Al/05/508
Av. de Beaumont 23, CH-1011 Lausanne
Tél. +4121 314 36 91/ Fax +41 21 314 48 87
borja.rodriguez-herreros@chuv.ch

Glossary

Connectomics: The study of the brain from a network perspective. The brain is represented as a graph, where nodes are different brain regions, and edges are functional or anatomical connections derived from MRI, EEG or other imaging methods. Here, we focus on anatomical connections derived from diffusion MRI.

Connectivity matrix: A matrix representing the brain network. Each row/column represents a brain region, and each entry is a connection property between a pair of brain regions. Entries in binary matrices are either 1 or 0 depending on whether an anatomical connection was reconstructed between the respective pair of brain regions or not. Weighted connectivity matrices are filled with metrics representing connectivity strength, or some other connectivity measures. Here, we focus on 85 cortical and subcortical regions taken from the Desikan-Killiany atlas. Connectivity weights are derived from a microstructure model that captures the fraction of restricted diffusion signal, which has been associated with the intra-axonal volume fraction.

Network metrics: Metrics derived from connectivity matrices, summarizing network properties at different levels. Global metrics are single values describing the overall network as a whole. Nodal metrics summarize node properties. Edge metrics are edge properties, which in our study is simply the fraction of intra-axonal volume allocated to each connection. Two small binary and weighted networks with 4 nodes (a, b, c, and d) and 4 edges are shown below, and are used to illustrate network metrics described hereafter (edge weights are labeled in blue):

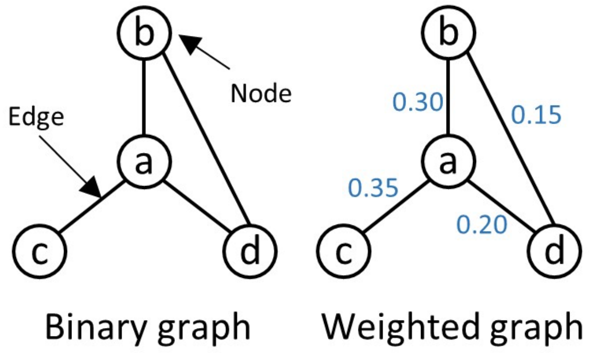

Degree: Nodal metric derived from a binary matrix, reporting the number of edges connected to a brain region (node). Measures how many brain regions a node is connected to. The following illustration shows that degree of node a is equal to 3. Degree of nodes b, c, and d are equal to 2, 1, and 2, respectively.

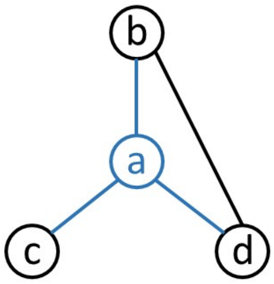

Degree = 3

Average degree: Global metric derived from a binary matrix, measuring the average number of edges per node. In the illustrative example, the node degrees are 2, 3, 1, and 2, which gives an average degree of 2.

Strength: Nodal metric derived from a weighted matrix, reporting the sum of edge weights connected to a node. In our study, this represents the intra-axonal volume fraction connecting a node with the rest of the brain. The following illustration shows that the strength of node a is equal to 0.30+0.35+0.20=0.85. The strength of nodes b, d, and d is 0.45, 0.35, and 0.35, respectively.

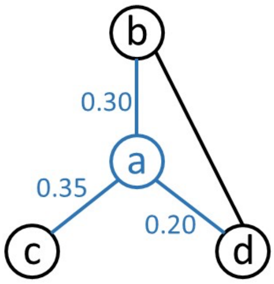

Strength = 0.85

Average strength: Global metric derived from a weighted matrix, measuring the average intra-axonal volume fraction allocated to connect a node to the rest of the brain. In our weighted graph example, the average strength is 0.50.

Clustering coefficient: Nodal metric reporting how much a node's neighbours are connected among themselves. For binary matrices, it measures the fraction of connections between neighbours with respect to all possible connections between themselves. The following illustration shows that the clustering coefficient of node a is equal to 1/3 (the edge connecting node neighbours is shown in blue, while unconnected neighbours have dashed blue links between them). Nodes b and d have a clustering coefficient of 1.0, while the clustering coefficient is not defined for node c as it only has one neighbor.

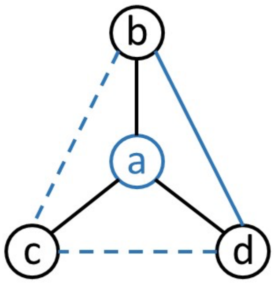

Clustering = 1/3

In our study, the weighted clustering coefficient represents the proportion of axonal volume used to connect neighbours that are connected between themselves, with respect to the total volume used to connect pairs of neighbors. In our illustrative weighted graph, neighbors of node a are connected through node a by a total volume of (0.30+0.35) + (0.30+0.20) + (0.35+0.20) = 1.7. As only node b and node d are connected directly between themselves, the weighted clustering coefficient is (0.30+0.20)/1.7=0.294 (i.e., almost 30% of the volume dedicated to connect neighbors through node a is used to connect neighbors that have a link between themselves). The weighted clustering coefficient for nodes b, and d is equal to 1.0 (they both have neighbors that are all connected between themselves). The weighted clustering coefficient for node c is not defined as it has only one neighbor.

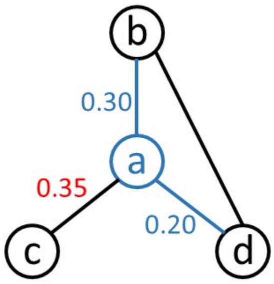

Clustering = 0.294

Global clustering coefficient: Global metric representing the average clustering coefficient. In our example networks, the global clustering coefficients are not defined because of node c.

Shortest path: For binary matrices, the path with lowest number of intermediate connections to link two nodes (the path length is the number of connections). The illustration below shows the shortest-paths in our example graph, annotated with the corresponding path lengths:

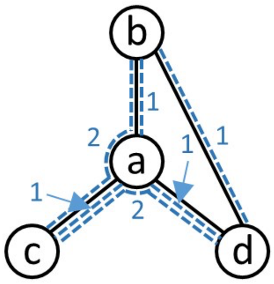

Shortest-paths

For weighted matrices, the path with lowest cumulative inverse intra-axonal volume fraction (the path length is the cumulative inverse volume). As larger volume is associated with higher connectivity strength, distance is taken as the inverse volume fraction. The following illustration shows the shortest-paths for our weighted graph, annotated with the respective path lengths:

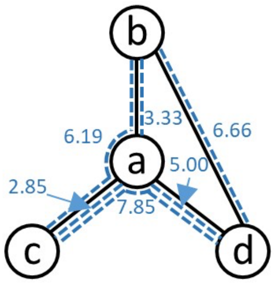

Shortest-paths

Node efficiency: Nodal metric measuring the average inverse shortest-path length required to transfer information from the node of interest to all other nodes in the network. For binary matrices, it measures the average inverse number of connections required to link a node to all other nodes. The following illustration shows that the efficiency of node b is equal to (1/1 + 1/2 + 1/1)/3=0.833. Similarly, the efficiency for nodes a, c, and d is 1.00, 0.67, and 0.83, respectively. Node a is the most efficient node as it is directly connected to all other nodes.

Node c is the less efficient node, as it is only directly connected to node a, and requires two edges to reach nodes b and d.

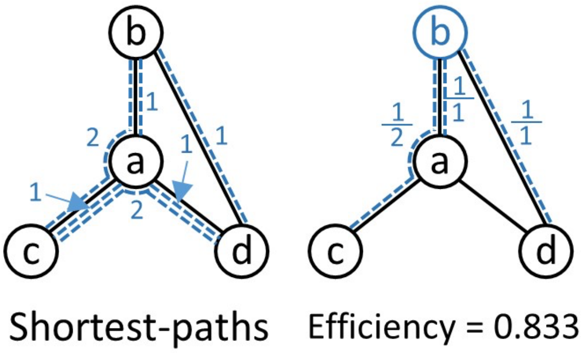

For weighted matrices, it measures the average inverse shortest-path length dedicated to connect a node to all other nodes in the network. The following illustration shows the weighted efficiency for our weighted graph example, which for node b is equal to (1/3.33+1/6.19+1/6.66)/3=0.204. Similarly, the weighted efficiency for nodes a, c, and d is 0.283, 0.213, and 0.15, respectively.

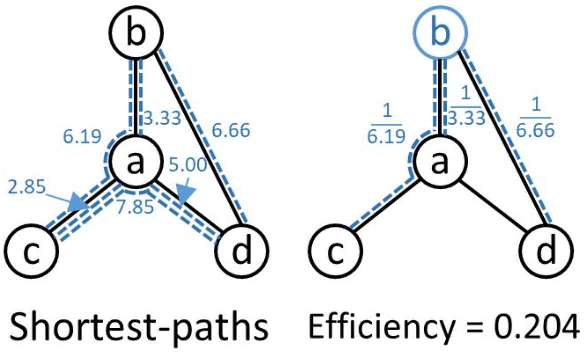

Global efficiency: Global metric reporting the average node efficiency in the network. In our example binary network, the global efficiency is 0.83, while the global efficiency for the weighted network is 0.215.

Node betweenness: Nodal metric representing the number of shortest-paths transiting through a node. The following illustration shows that betweenness for node a is equal to 2 (SPs: shortest-paths). The same betweenness is found for node a in the weighted network example.

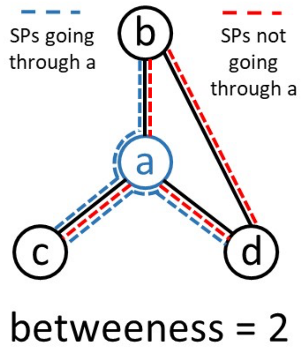

<u>Global betweenness</u>: The average node betweenness in the network.

<u>Node closeness</u>: Node metric measuring the inverse of the sumed shortest-path lengths (see definition of binary and weighted shortest-paths above) linking a node to all other nodes in the network. The following illustration shows that closeness of node c is equal to 1/(2+1+2)=0.20. Closeness for nodes a, b, and d are 0.33, 0.25, and 0.25, respectively.

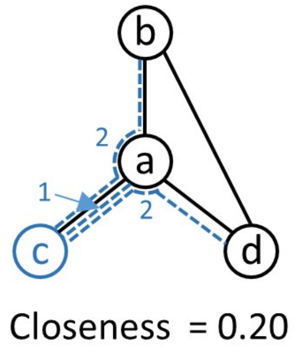

The following illustration shows that closeness for node c in the weighted network example is equal to 1/(6.19+2.85+7.85)=0.06. Similarly, weighted closeness for nodes a, b, and d is equal to 0.089, 0.062, and 0.051, respectively.

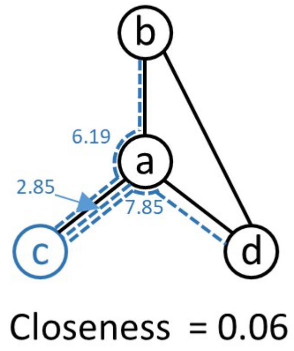

Global closeness: Global metric reporting the average node closeness of the network. In our illustrative binary graph, the global closeness is equal to 0.258, while the weighted graph has a global closeness of 0.065.

Global diameter: Global metric reporting the longest shortest-path in the network. In the illustrative graph, the global diameter is equal to 2 (longest shortest-paths are labeled in blue, while shorter ones are labeled in red):

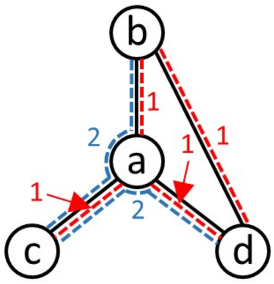

Diameter = 2

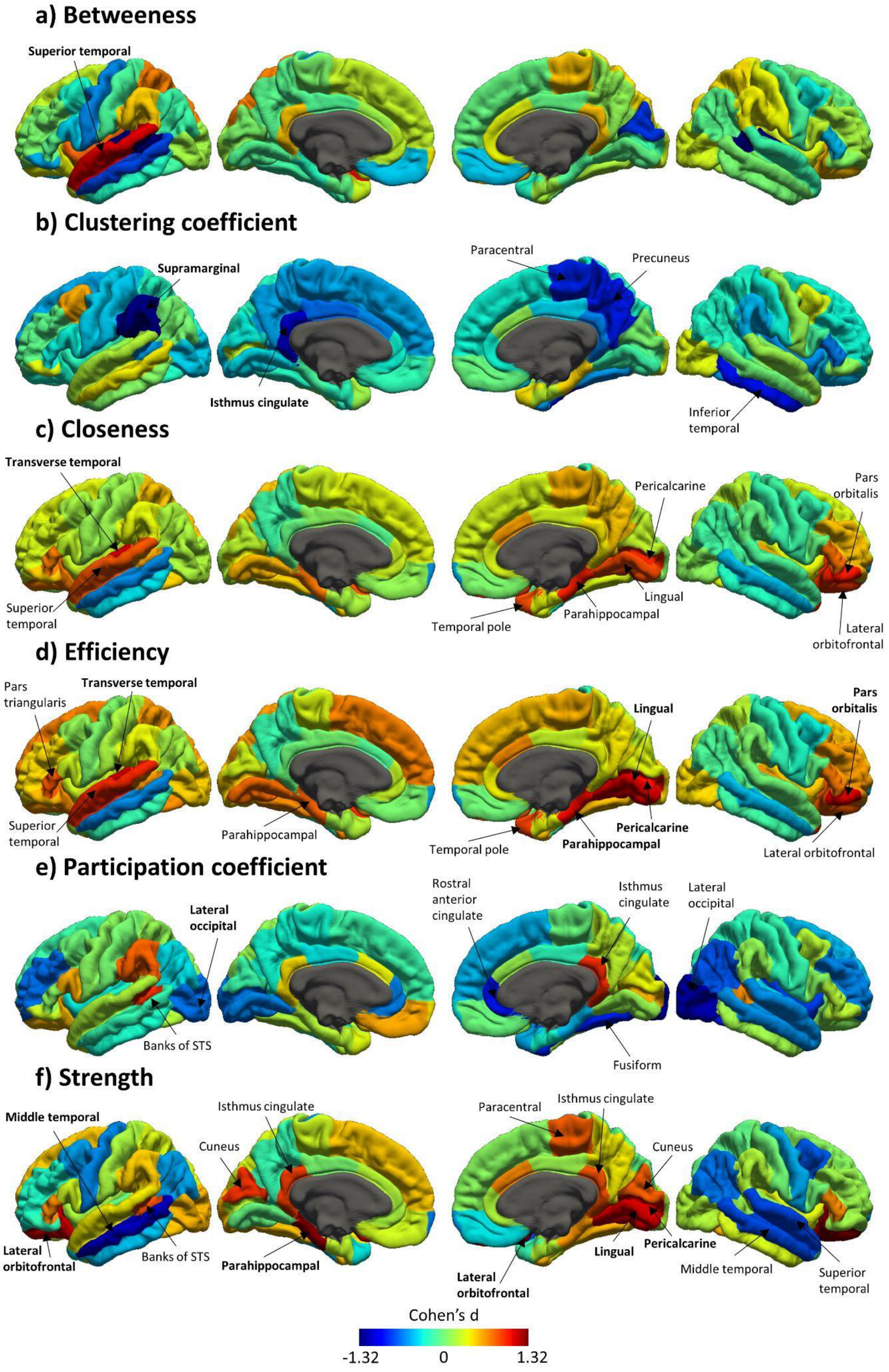

Supplementary Figure S1. Significant topological differences based on weighted connectivity matrices. a) Strength, b) efficiency, c) clustering coefficient, d) closeness, e) betweenness, and f) participation coefficient. In each panel, cortical regions are shown on the pial surface of Freesurfer's average subject, colored according to effect size (Cohen's d). Regions surviving Bonferroni correction are labeled in bold, while regions surviving FDR corrections are labeled in standard font.

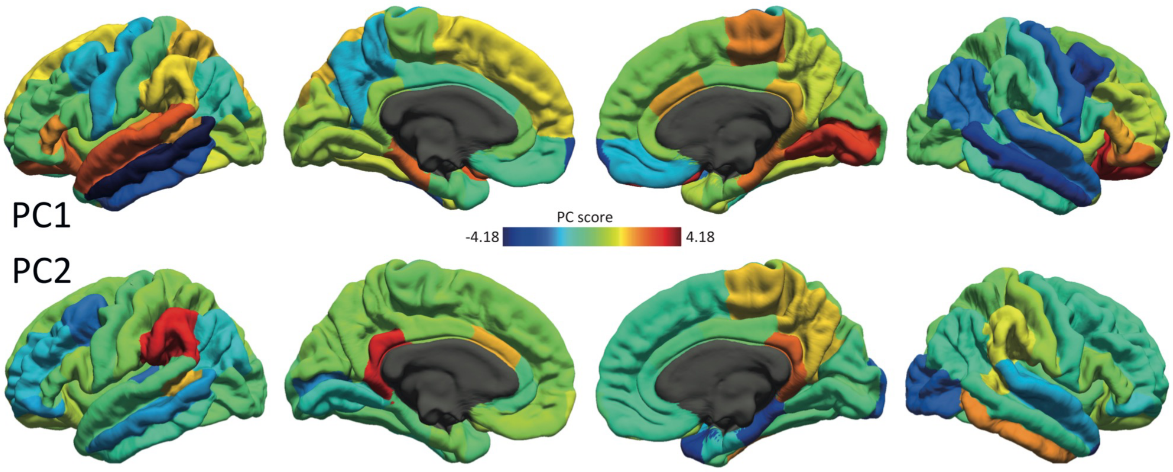

Supplementary Figure S2. Cortical representation on the pial surface of the PC1 (upper row) and PC2 (lower row) scores for each brain region.
